# Supplementary material for: Effectiveness and Safety of Ayurvedic Medicines in Type 2 Diabetes Mellitus Management: A Systematic Review and Meta-Analysis
Source: Front Pharmacol. 2022 Jun 8;13:821810. doi: 10.3389/fphar.2022.821810 (PMC9213670; doi:10.3389/fphar.2022.821810)
Supplement: Supplementary file 1 [file DataSheet1.zip › Supplementary Material/Forest and Funnel Plots/Trigonella foenum-graecum (versus OAD)/HbA1c.pdf]

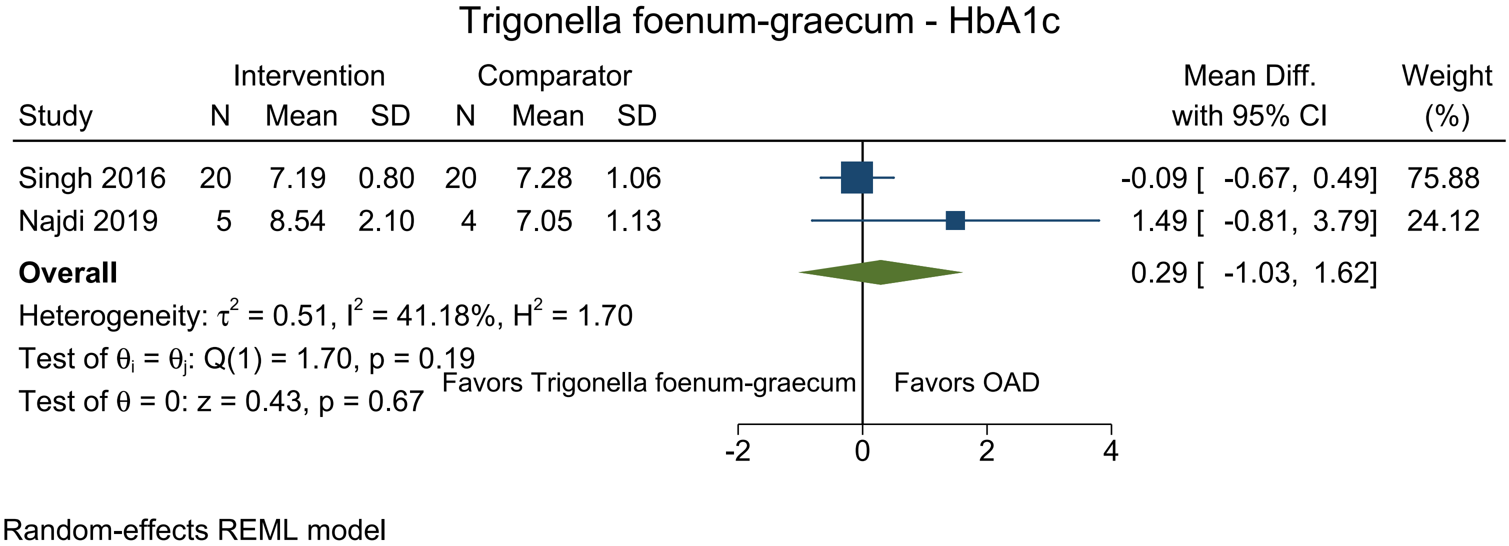
Trigonella foenum-graecum - HbA1c
Intervention
Comparator
Study
N
Mean
SD
N
Mean
SD
Mean Diff. with 95% CI
Weight (%)
Singh 2016 20 7.19 0.80 20 7.28 1.06 -0.09 [-0.67, 0.49] 75.88
Najdi 2019 5 8.54 2.10 4 7.05 1.13 1.49 [-0.81, 3.79] 24.12
Overall 0.29 [-1.03, 1.62]
Heterogeneity: $\tau^2 = 0.51$, $I^2 = 41.18\%$, $H^2 = 1.70$
Test of $\theta_i = \theta_j$: Q(1) = 1.70, p = 0.19
Test of $\theta = 0$: z = 0.43, p = 0.67
Favors Trigonella foenum-graecum
Favors OAD
-2
0
2
4
Random-effects REML model
